# Systems Modeling of Interactions between Mucosal Immunity and the Gut Microbiome during *Clostridium difficile* Infection

**DOI:** 10.1371/journal.pone.0134849

**Published:** 2015-07-31

**Authors:** Andrew Leber, Monica Viladomiu, Raquel Hontecillas, Vida Abedi, Casandra Philipson, Stefan Hoops, Brad Howard, Josep Bassaganya-Riera

**Affiliations:** 1 The Center for Modeling Immunity to Enteric Pathogens, Virginia Bioinformatics Institute, Virginia Tech, Blacksburg, Virginia, United States of America; 2 Nutritional Immunology and Molecular Medicine Laboratory (www.nimml.org), Virginia Bioinformatics Institute, Virginia Tech, Blacksburg, Virginia, United States of America; 3 Department of Biological Sciences, Virginia Bioinformatics Institute, Virginia Tech, Blacksburg, Virginia, United States of America; Charité, Campus Benjamin Franklin, GERMANY

## Abstract

*Clostridium difficile* infections are associated with the use of broad-spectrum antibiotics and result in an exuberant inflammatory response, leading to nosocomial diarrhea, colitis and even death. To better understand the dynamics of mucosal immunity during *C*. *difficile* infection from initiation through expansion to resolution, we built a computational model of the mucosal immune response to the bacterium. The model was calibrated using data from a mouse model of *C*. *difficile* infection. The model demonstrates a crucial role of T helper 17 (Th17) effector responses in the colonic lamina propria and luminal commensal bacteria populations in the clearance of *C*. *difficile* and colonic pathology, whereas regulatory T (Treg) cells responses are associated with the recovery phase. In addition, the production of anti-microbial peptides by inflamed epithelial cells and activated neutrophils in response to *C*. *difficile* infection inhibit the re-growth of beneficial commensal bacterial species. Computational simulations suggest that the removal of neutrophil and epithelial cell derived anti-microbial inhibitions, separately and together, on commensal bacterial regrowth promote recovery and minimize colonic inflammatory pathology. Simulation results predict a decrease in colonic inflammatory markers, such as neutrophilic influx and Th17 cells in the colonic lamina propria, and length of infection with accelerated commensal bacteria re-growth through altered anti-microbial inhibition. Computational modeling provides novel insights on the therapeutic value of repopulating the colonic microbiome and inducing regulatory mucosal immune responses during *C*. *difficile* infection. Thus, modeling mucosal immunity-gut microbiota interactions has the potential to guide the development of targeted fecal transplantation therapies in the context of precision medicine interventions.

## Introduction

*Clostridium difficile*, a Gram-positive spore-forming, anaerobic bacterium, often colonizes the human gastrointestinal tract after disruption of the normal intestinal flora. *C*. *difficile* infection (CDI) is a leading cause of diarrhea and pseudomembranous colitis in hospital acquired infection due to prolonged doses of antibiotics [1]. Based on a study published in 2015, the occurrence rate of *C*. *difficile* in the United States is 147 cases per 100,000 people. Additionally, *C*. *difficile* is estimated to be responsible for 29,000 deaths per year, a 50% increase from the 2007 estimate of 14,000 [2, 3]. The rate of *C*. *difficile*-associated infections and deaths may be rising due to the emergence of the hyper virulent strains that exhibit resistance to traditional fluoroquinolone antibiotics [4]. Paradoxically, standard treatment of *C*. *difficile* associated disease (CDAD), an illness linked to antimicrobial usage, includes administration of more antibiotics such as metronidazole or vancomycin. Indeed, this therapeutic approach may contribute to the considerable rates of recurrence, estimated to be between 5 to 30% [5]. Recently, alternative strategies have been devised to decrease rates of recurrence through use of toxin neutralizing antibodies or gut microbiome reconstitution through fecal transplantation [6, 7]. Beyond the implications in CDI, the gut microbiome has also been implicated as a predictor of autoimmune and inflammatory diseases such as inflammatory bowel disease (IBD) as well as obesity [8]. The ability to functionally evaluate the impact of host-microbiota interactions on health outcomes could guide the use of reconstitution therapies for the treatment of a wide range of human diseases. Additionally, the *baiCD* gene, which encodes a bile acid processing enzyme, allows commensal microbes to utilize host-produced bile salts to synthesize metabolites, such as deoxycholate and lithocholate that provide resistance against *C*. *difficile* [9].

CDI is most often predicated by an alteration in commensal bacteria, usually as a result of prolonged administration of broad-spectrum antibiotics [10]. The decrease or removal of competitive species allows the vegetative *C*. *difficile* in the intestinal lumen to proliferate. In accordance with the increased population, there is enhanced production and release of the two toxins, TcdA and TcdB, thought to be the main virulent factors in CDI. TcdA is an enterotoxin which causes tissue damage and edema as a result of interaction with luminal epithelial cells [11]. The disruption of the epithelium by TcdA facilitates the migration of TcdB into the lamina propria layer where it triggers multiple immune response mechanisms. For instance, TcdB can directly interact with monocytes causing a shift in macrophage populations to an M1 phenotype and an increase in the concentration of pro-inflammatory cytokines [12]. The pro-inflammatory environment combines with the effects of TcdA to alter the state of epithelial cells leading to the activation and migration of neutrophils into the intestinal lumen [13]. Specifically, the inflamed epithelial cells have exhibited increased secretion of interleukin-8 (IL-8), an important cytokine in the creation of a gradient inducing neutrophil chemotaxis [14] and a biomarker of severity of disease. The migration of and subsequent release of cytotoxic granules from neutrophils reduces the pathogen number while also further damaging the epithelium. The presence of pathogenic *C*. *difficile* also increases accumulation of effector dendritic cells (DC) as a result of increased contact rates between the pathogen and immature DC sampling the lumen, and the increased engulfment rate of the bacteria [15]. The prevalence of effector DC induces Th1 and Th17 effector responses [16]. These subsets of CD4+ T helper cells favor a pro-inflammatory environment in the colonic lamina propria (LP). To modulate the inflammatory microenvironment, immature DC are stimulated to become tolerogenic DC which induce the differentiation of naïve CD4+ T cells into induced regulatory T helper (Treg) cells. For instance, activation of PPARγ, also contributes to a production of Treg cells from fully differentiated Th17 cells, adding a second source pathway through mechanisms of plasticity [17]. Treg responses suppress mucosal inflammation through deactivation of effector dendritic cells, suppression of Th1 responses, and the shift of Th17 into Treg subsets [18]. Failure to mount this regulatory response may worsen symptoms and expected infection outcome. Our modeling approaches have examined how impaired regulatory responses may influence pathology and disease.

Computational modeling has shown promise in integrating theory, procedural knowledge, and data for capturing the experimental observations in synthetic information processing systems and predicting emerging behaviors. Recently, models have been developed in the context of bacterial or viral infection and inflammatory diseases [19–21]. Additionally, the involvement of various cell types across varying dynamic patterns in mucosal immune responses requires improved methodology to understand complex and massively interacting host-microbiota networks. Through the creation of a computational host-microbiome network, the merging of computational and in vivo experimental immunology approaches offers the ability to analyze the microbiome-driven changes in the gut immune cell composition that have been shown to extend to a systemic alteration of the immune environment in the context of multiple diseases [22]. Once calibrated and validated, these models become valuable tools to generate novel hypotheses and guide the design of innovative non-intuitive experiments *in vivo*. In addition, the validated models could contribute to the development of microbiome-based therapeutics for the prevention and amelioration of disease.

It has been shown that a shift towards a pro-inflammatory phenotype, specifically a Th17-driven response with a decreased Treg cell population occurs due to a lack of PPARγ in CD4 T cells, leading to increased disease symptoms and colonic pathology during CDI [23]. To further understand these cellular dynamics and the overall host response to the bacterium, we have generated a tissue-level computational model of *C*. *difficile* infection using ordinary differential equations (ODEs) to describe the experimentally observed dynamics from individual cells to the gut mucosal immune system. In addition to the immune response, the model incorporates the interactions between host, pathogen and commensal bacteria and can be customized to model other bacteria and associated conditions. Modeling results illustrate the relative impacts of regulatory and effector components of the mucosal immune response in the clearance of *C*. *difficile* and damage to the colonic mucosa. Model predictions highlight the role of the host microbiome re-growth in controlling the immune response and CDI dynamics at the colonic mucosa.

## Results

### Modeling mucosal immune responses to *Clostridium difficile* infection

**Fig 1:**
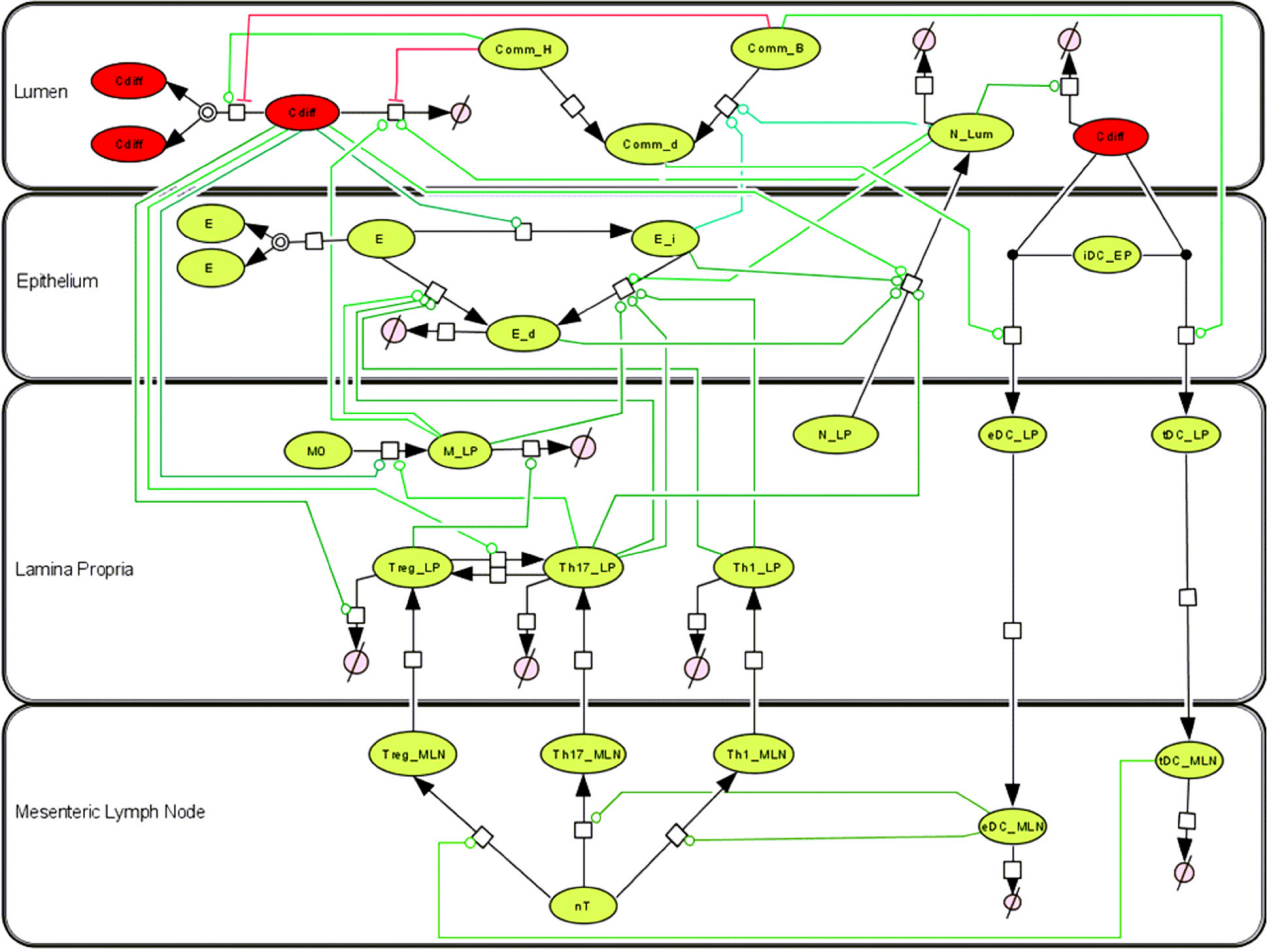
Network topology of model illustrating mucosal immune responses to *Clostridium difficile*. Systems biology markup language (SBML) compliant network of interactions between *C*. *difficile* and cellular immune components created in CellDesigner. Reaction modifiers connect cell nodes to reaction arrows with green as indication of activation and red of inhibition. Species consist of *C*. *difficile* (*Cdiff*), infection-exacerbating commensal bacteria (*CommH*), protective commensal bacteria (*CommB*), dead commensal bacteria (*CommD*), epithelial cells (*E*), inflamed epithelial cells (*E*_*i*_), neutrophils (*N*), macrophages (*M*), dendritic cells (*tDC* and *eDC*), T cells (*nT*, *Treg*, *Th17*, *Th1*) existing in multiple compartments lumen (*Lum*), epithelium (*EP*), lamina propria (*LP*), and mesenteric lymph node (*MLN*).

The model topology is shown in Fig 1. The network represents the immune response to CDI in the colonic mucosa. The *C*. *difficile* immune response model incorporates three reactions for the *C*. *difficile* species (*Cdiff*), which are activated or inhibited by seven host or gut microbiota modifiers. The *Cdiff* species may proliferate. The proliferation reaction is activated by the infection-exacerbating commensal species, *CommH*, and inhibited by the protective commensal species, *CommB*. The *Cdiff* species may also die, in which the *Cdiff* population is reduced. The latter is triggered by the activated macrophage species, *M*, as well as the activated neutrophil species, *N*_*Lum*_. *Cdiff* death is also inhibited by the *CommH* species. Furthermore, *Cdiff* may interact with a dendritic cell, *iDC*_*Ep*_, to produce an activated dendritic cell, with the effector and tolerogenic balance regulated by the comparative population of *CommB* to the dead commensal species, *CommD*. In addition, the *Cdiff* species acts as a modifier of five reactions: the inflammation of colonic epithelial cells, the activation and migration of neutrophils, the activation of macrophages, the death of Treg cells, and the plasticity between Treg and Th17 CD4+ T cell subsets. The computational model is comprised of four compartments (lumen, epithelium, lamina propria and mesenteric lymph nodes) and 23 species whose interactions are described by 30 reactions. The resulting ordinary differential equations (ODE) utilize 49 parameters to describe the dynamics of the system. Parameter values were determined using data generated through a murine model of CDI.

### Kinetics of *Clostridium difficile-*induced CD4 T cell responses in mice

**Fig 2:**
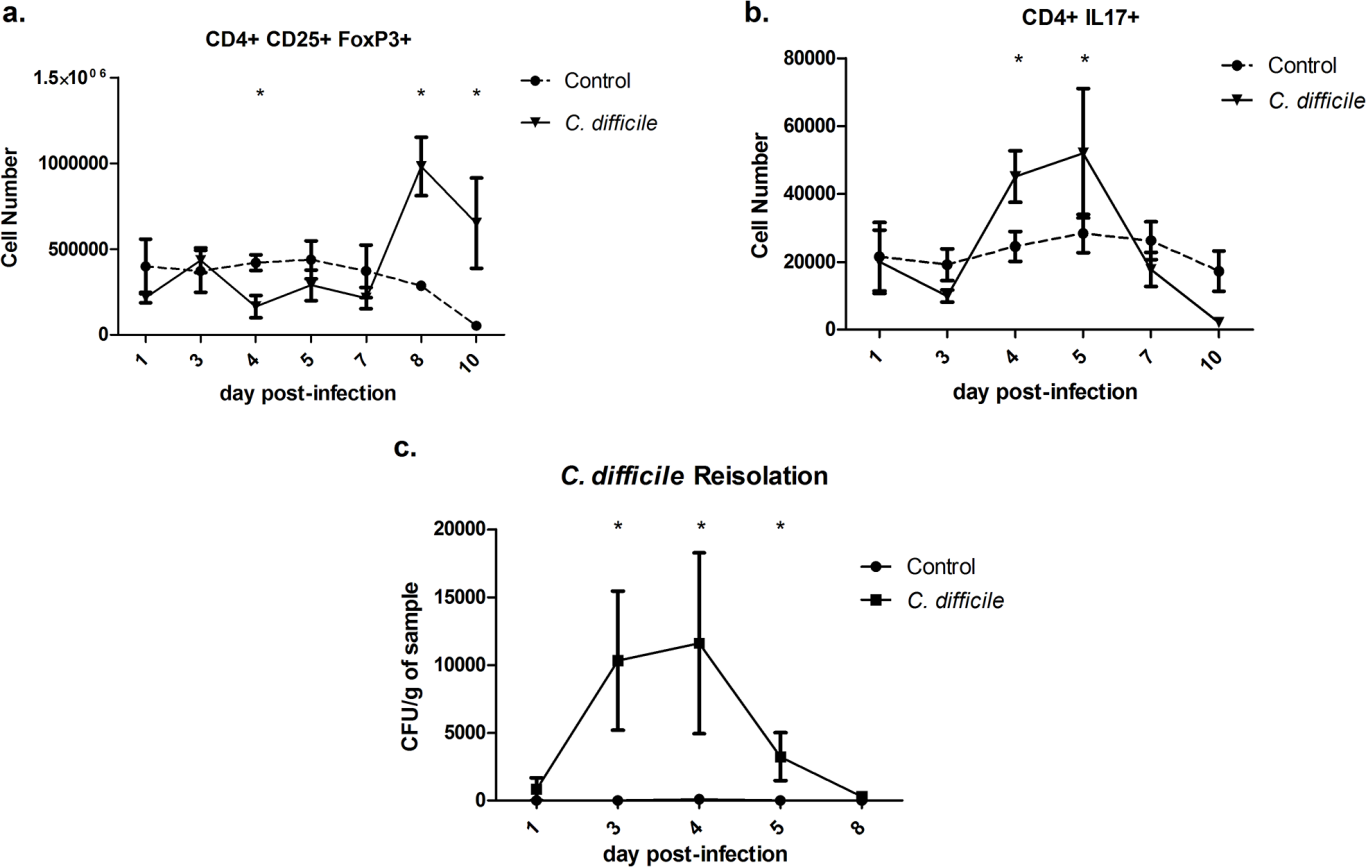
Time course of *Clostridium difficile* infection in mice. (a and b) Flow cytometry analysis of colonic lamina propria lymphocytes from days 1 to 10 post-infection showing the differences in CD4+ CD25+ FoxP3+ regulatory T (Treg) and CD4+ IL17+ T helper 17 (Th17) cells, respectively, between control and *C*. *difficile* challenged wild type mice. (c) Re-isolation data of *C*. *difficile* from colonic contents from day 1 to day 8 post-infection. Data points and error bars represent mean ± standard error of the mean (SEM). Asterisks (*) mark significance (*P*≤0.05) in comparison between control and *C*. *difficile* infected mice (n = 10).

**Fig 3:**
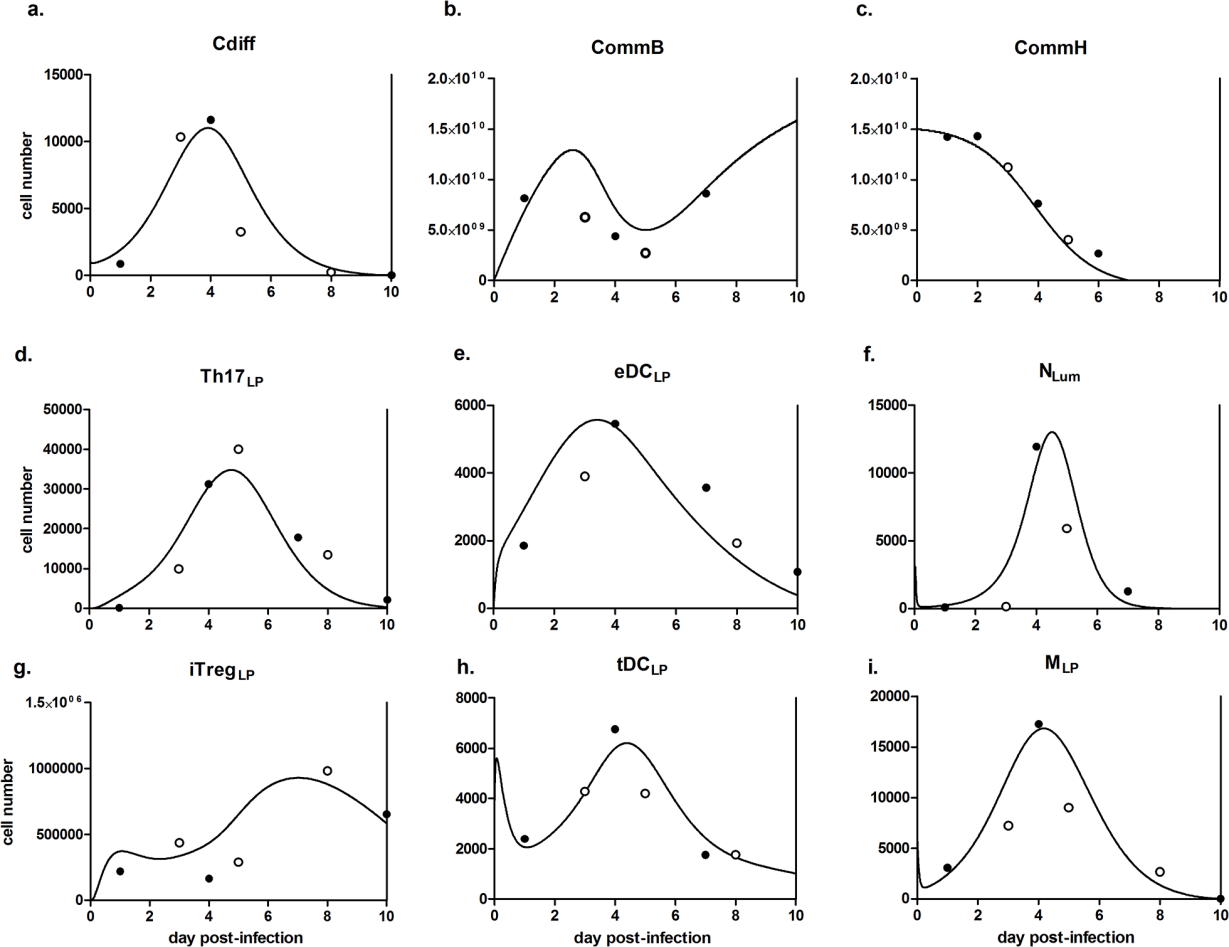
Simulated dynamics of mucosal immune response to *Clostridium difficile*. Modeling results following calibration and validation of the host response model in populations of (a) *C*. *difficile*, (b) protective commensal bacteria, (c) infection-exacerbating commensal bacteria, (d) lamina propria T helper 17 cells, (e) effector dendritic cells, (f) infiltrating neutrophils, (g) regulatory T cells, (h) tolerogenic dendritic cells and (i) activated macrophages. Lines represent simulation results, filled points represent experimental calibration data and unfilled points represent experimental validation data.

A time course study was performed to evaluate changes in immune cell composition following CDI. On days 1, 3, 4, 5, 7, 8, and 10 post-infection, colons and MLN were collected, processed and assayed to determine alterations in immune cell subsets by flow cytometry conducted in two sets. In addition, colonic contents were collected and plated to measure the *C*. *difficile* population size. The response to infection was observed to be Th17 dominant with a large neutrophilic influx in the colonic mucosa. Chronologically, the pro-inflammatory response, marked by an increase in the Th17 effector cell subset, was initiated between days 3 and 4 post-infection (Fig 2B). The Th17 response peaked on day five post-infection and corresponds with the largest increase in macrophage accumulation. In contrast a regulatory response, characterized by the accumulation of CD4+CD25+Foxp3+ Treg cells was detected between days 8 and 10, and preceded by a slight suppression between days 4 and 7 post-infection (Fig 2A). Bacterial re-isolation from colonic contents displayed that the peak of the *C*. *difficile* population occurred on day 4 post-infection and the bacterium was largely cleared by day 8 (Fig 2C). The data displays that CD4+ T cells may crucially contribute to the response to CDI. For this reason, these populations were foundational elements of the computational model. Combined, this data displays that while the peak of the *C*. *difficile* population occurs on day 4 post-infection, important events continue to occur through day 10 post-infection. The results established key time points during infection for the evaluation of simulation results such as the peak of *C*. *difficile* population between days 3 and 4, of the inflammatory response between days 4 and 5, and of the regulatory response between days 8 and 10. Additional data from Buffie, *et al*. was used to calibrate the commensal populations with 16S analysis of the host microbiome regrowth following antibiotic ablation [9]. Data from Blake, *et al*. was used in combination with generated data to calibrate neutrophil populations [24]. Epithelial cells were calibrated with a combination of previously reported data [25, 26]. The data from two time courses of infection was compiled into separate calibration and validation datasets. The calibration dataset includes data gathered during the first experiment on days 1, 4, 7 and 10 post-infection as well as further sourced data from intermediate days. The validation dataset includes data generated on days 3, 5 and 8 post-infection. Parameters for the computational model were estimated using Particle Swarm and Genetic algorithms as implemented in COPASI [27]. The calibrated ODE model is able to replicate the dynamics observed in the time course of infection shown in Fig 3.

### Sensitivity analysis helps identify determinants of *C*. *difficile* population control and epithelial damage

**Fig 4:**
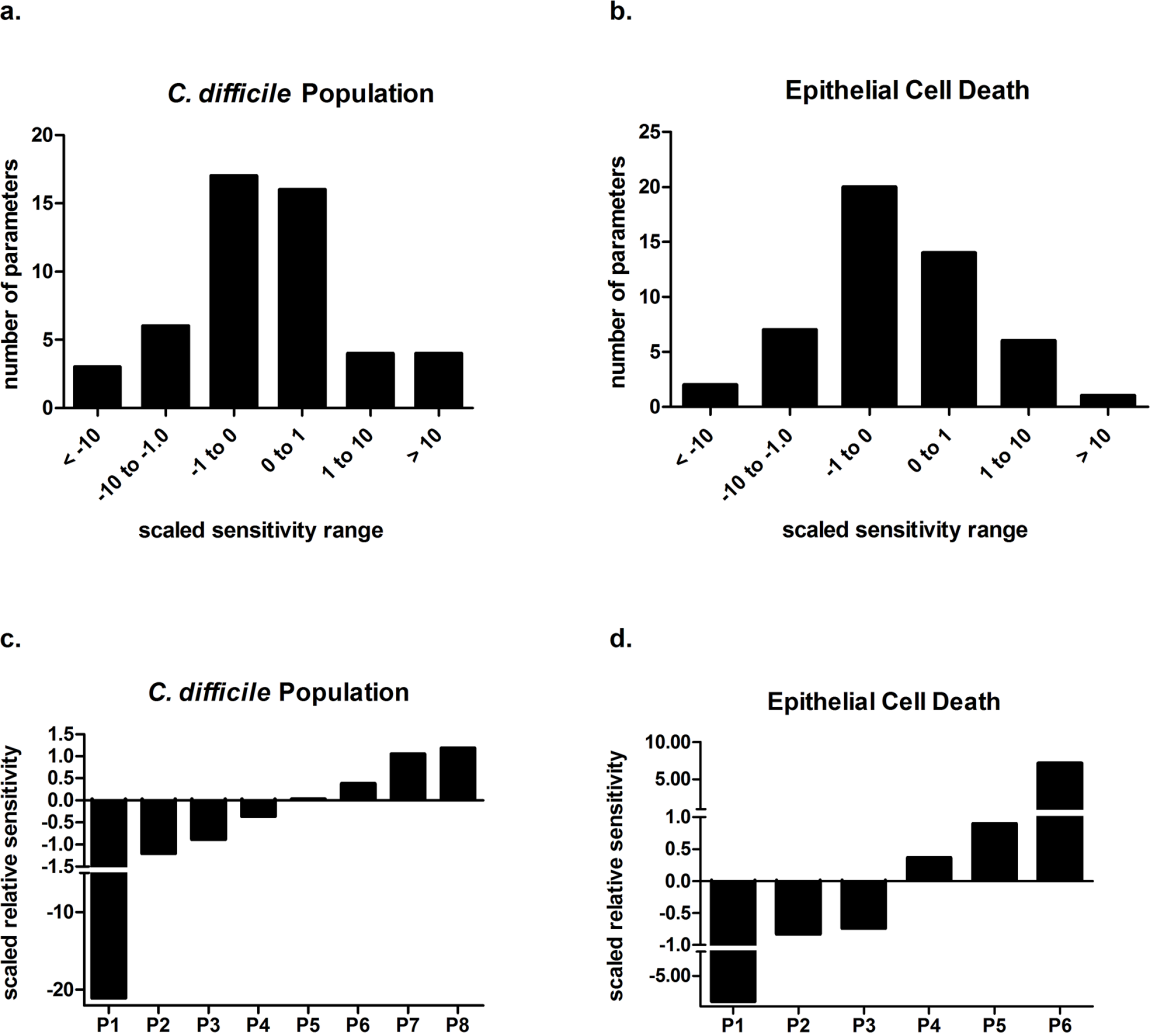
Relative effects of parameters on *Clostridium difficile* population and epithelial cell death. (a and b) Histograms showing the distribution of parameter impact on *C*. *difficile* population and epithelial cell death, respectively. Measurements are based on sensitivity analysis of the calibrated model. (c and d) Highest impact parameters for each quantity in which positive amounts indicate an increasing effect on the quantity and negative amounts indicate a decreasing effect. For the *C*. *difficile* population results, P1 contributes to effector dendritic cell production, P2 to neutrophil activation and migration, P3 to protective commensal bacteria regrowth, P4 to macrophage activation, P5 to commensal bacteria death, P6 to macrophage death, P7 to Th17 cell death, and P8 to neutrophil death. For epithelial cell death, P1 contributes to tolerogenic dendritic cell production, P2 to Th17 to Treg cell plasticity, P3 to commensal bacteria death, P4 to Treg to Th17 cell plasticity, P5 to macrophage activation and P6 to *C*. *difficile* growth.

The clearance of *C*. *difficile* and colonic epithelial damage are two interconnected end effects that contribute to the overall infection severity. Sensitivity analysis on the computational model was used to determine which parameters in the network had the greatest influence on these factors. Each quantity has a distribution of parameter impacts with a large number of low impact parameters centered around zero, and smaller amounts with both positive and negative effects at larger magnitudes (Fig 4A and 4B). In each case the very large impact parameters are, by majority, directly involved with the population being analyzed. Because of this direct relation, little can be gained beyond what is naturally intuitive from delving into these parameters further. As a result, these parameters were not evaluated further. Parameter 5 through 8 in Fig 4C were associated with an increase in the *C*. *difficile* population in the lumen and included the degradation and death rates of effector immune cells, macrophages, neutrophils and Th17 cells, as well as the death rate of commensal species. In contrast, the production of effector DC, parameter 1, greatly contributed to a decrease in the *C*. *difficile* population. The impact of balancing regulatory and effector arms of the mucosal immune response is also displayed through effects on epithelial cell death (Fig 4D). The induction of the Treg cell response through the production of tolerogenic DC (P1), plasticity with Th17 cells (P2) and a regrowth of commensal bacterial species (P3) possess strong decreasing effects on the amount of epithelial cell death.

### Increased production of anti-microbial peptides by epithelial cells exerts an inhibitory effect on beneficial commensal microbiota species

**Fig 5:**
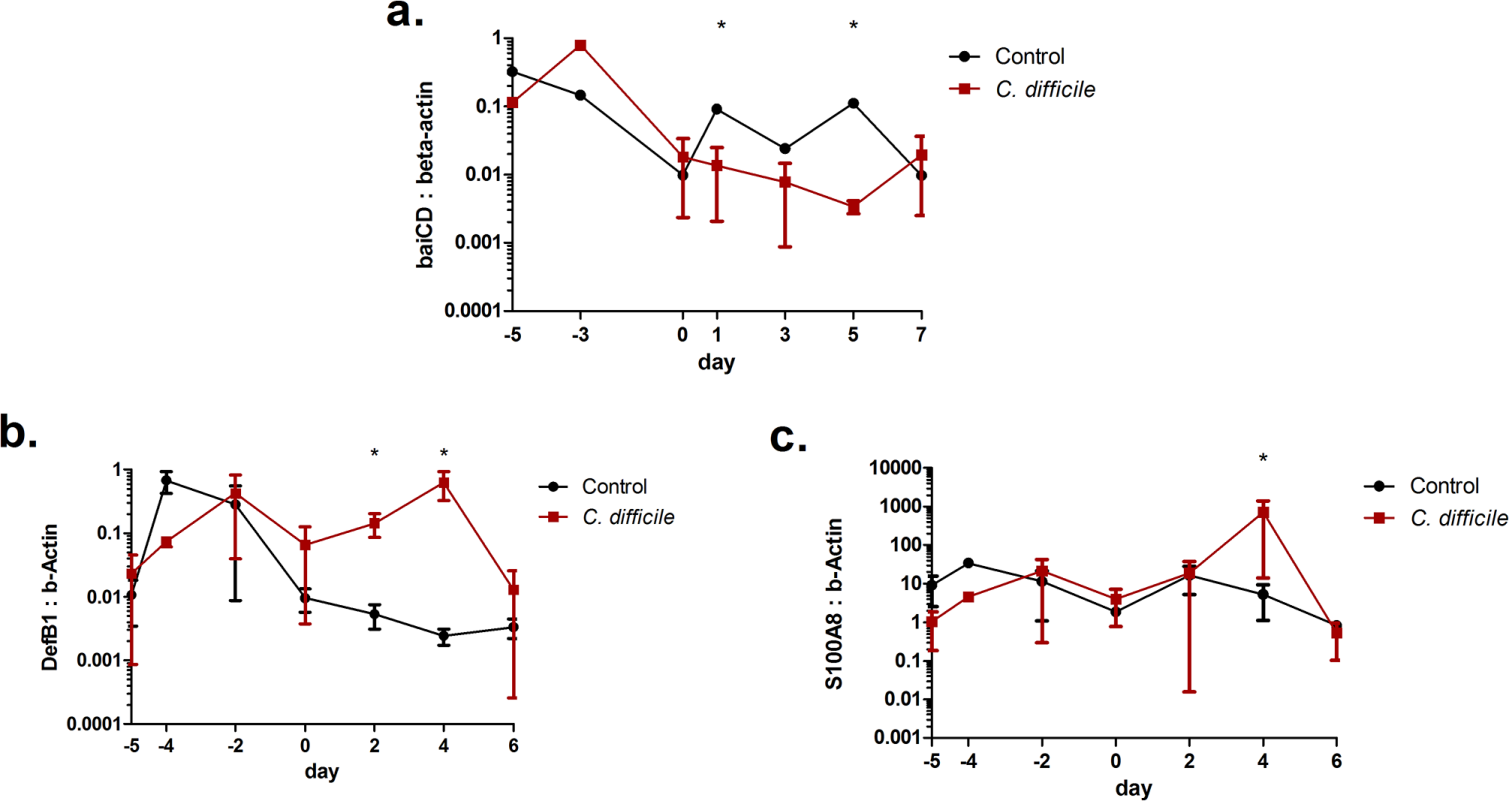
Commensal bacteria regrowth inhibited with *Clostridium difficile* infection. (a) The *baiCD* content is decreased by antibiotic treatment in both control and *C*. *difficile* challenged mice and further decreased post-infection in the *C*. *difficile* challenged mice compared to the controls. (b and c) The expression of anti-microbial peptides DefB1 and S100A8 are upregulated with infection. Data points and error bars represent mean ± standard error of the mean (SEM). Asterisks (*) mark significance (p≤0.05) in comparison between control and *C*. *difficile* infected mice (n = 10).

Quantitative RT-PCR analyses conducted on colonic contents demonstrated a difference in commensal species regrowth between *C*. *difficile* challenged and control mice (Fig 5A). After intraperitoneal injection of clindamycin, amount of the *baiCD* gene is reduced 1.5 orders of magnitude on day zero compared to the initial amount on day 5 pre-infection, showing a dramatic reduction of protective commensal species after administration of antibiotics. Additionally, prior to challenge, there is no significant difference between the two experimental groups. The control mice quickly recover nearly one-third of *baiCD* containing microbiota levels as early as day one with slight fluctuations around that point for the remainder of the time course. After *C*. *difficile* challenge, the *baiCD* containing microbiota levels continue to decrease through day 5 post-infection. The level begins to rebound to pre-challenge amount by day seven. The difference between the *C*. *difficile* challenged and control levels of *baiCD* content suggest that a *C*. *difficile*- or immune-mediated effect is present during CDI that prevents the regrowth of protective commensal species. The colonic expression of anti-microbial peptides, DefB1 and S100A8, was assayed by using qRT-PCR. The expression of DefB1 is significantly upregulated on days 2 and 4 post-infection in colonic contents of *C*. *difficile* infected mice before returning to control level on day 6 (Fig 5B). Concurrently, the expression of S100A8 in the *C*. *difficile* challenged mice followed that of the uninfected controls with the exception of a significantly upregulated peak on day 4 post-infection (Fig 5C).

### Removal of commensal bacteria regrowth inhibition alters host response to infection

**Fig 6:**
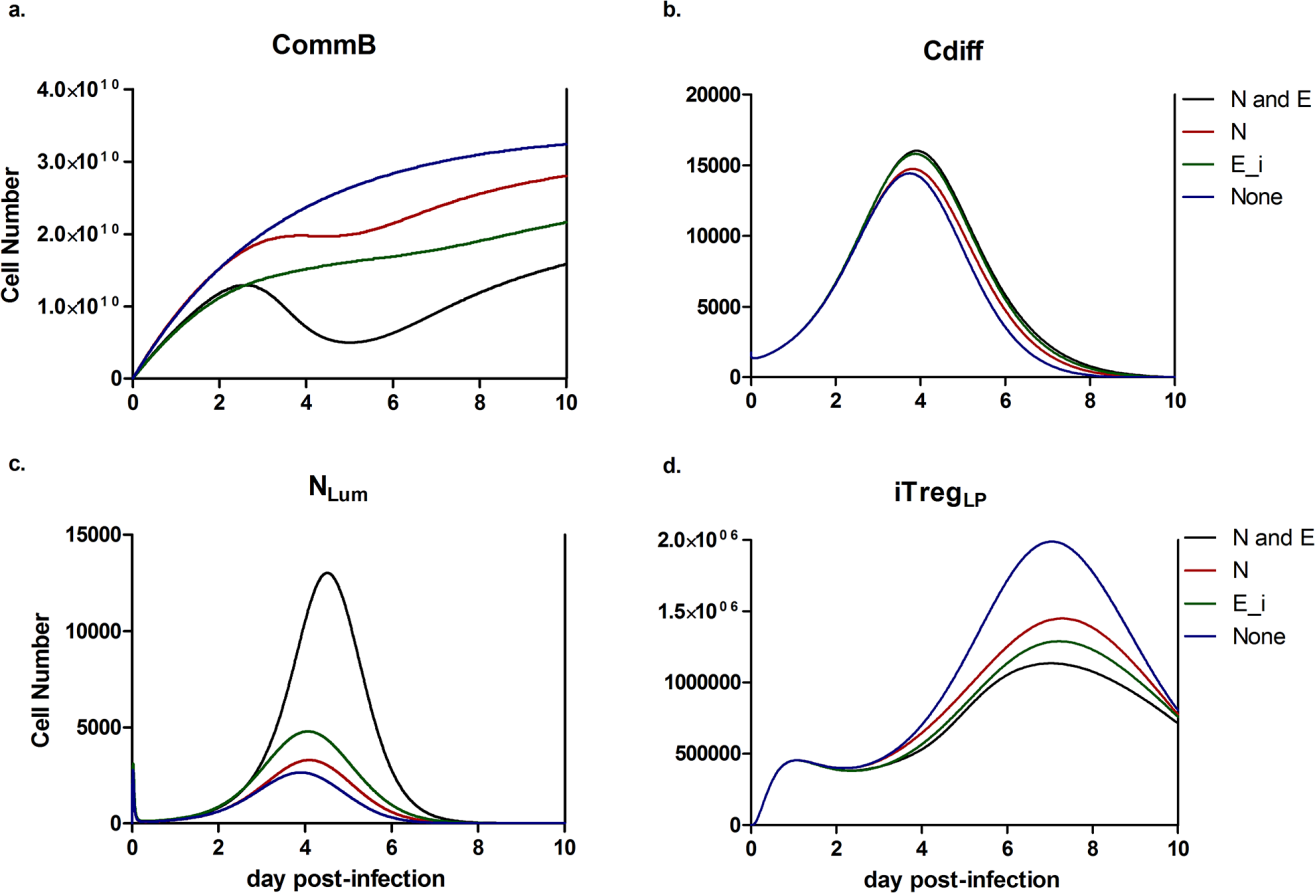
*In silico* simulation of altered commensal bacteria regrowth during *Clostridium difficile* infection. Four cases were tested with variations to the inhibition of the commensal bacteria growth: inhibited by both neutrophils and inflamed epithelial cells (N and E_i), by only neutrophils (N), by only inflamed epithelial cells (E_i), and by neither (none). Resulting changes in species populations for each case are shown: (a) *baiCD*-containing commensal species, (b) *C*. *difficile*, (c) activated neutrophils, and (d) iTreg cells in the lamina propria.

To display the effect of neutrophil and epithelial cell-derived anti-microbial peptides on the regrowth of commensal species, four scenarios were considered. A simulation exists for the combined inhibition by neutrophils and inflamed epithelial cells (*NE*), by only neutrophils (*N*), by only inflamed epithelial cells (*E*) and by neither. In both the *NE* and *E* simulations, the initial regrowth of beneficial commensal species, within the first two days post-infection, is slowed compared to the uninhibited case (Fig 6A). In comparison, the *N* simulation follows a similar pattern to the uninhibited case over the same time period. The *NE* simulation displays a reduction in beneficial commensal species following the initial stage and continuing through day 5 post infection. This reduction and deviation from regrowth of *baiCD* content on day 5 is representative of the *in vivo* data displayed in Fig 5A. The *NE* simulation further mimics the *in vivo* situation with a clear commitment to regrowth from day 5 to day 7. The *N* simulation has a similar trend to a smaller degree while the uninhibited and *E* simulations show continuation of their respective initial trends. In the final stage of the simulation, the *N* and *NE* cases return to increasing trends. The regrowth begins to slow in the uninhibited case and has an even greater deceleration in the *E* simulation. The *E* simulation showed little change in the *C*. *difficile* population at the peak of infection and a delayed clearance (Fig 6B) compared to the *NE* simulation. Both the *N* and uninhibited simulations displayed small reductions of *C*. *difficile* at the peak of infection relative to the *NE* case; however, only the uninhibited case cleared the *Cdiff* species by an earlier time point. The three altered simulations had reductions in peak neutrophil activation and influx compared to the *NE* case as well as occurring at slightly earlier times (Fig 6C). Each simulation possessed a larger iTreg peak in comparison to the *NE* case prior to converging to a similar resolution (Fig 6D). Only the uninhibited simulation greatly altered the timing of the peak in the iTreg response with an advance of approximately one half of a day.

## Discussion

CDI is a rising problem in the health care system. The standard treatment of infection is the discontinuation of any previous antibiotics and the administration of a new antibiotic regime. While the treatment is effective in certain cases, it can also result in significant rates of re-occurrence of CDI starting from a baseline of 25% [28]. Currently, numerous methods of alleviation of these treatment methods are under investigation, including the development of vaccines, toxin-based antibodies and microbiome reconstitution [7, 29, 30]. In this study, we used computational modeling in combination with immunology experimentation *in vivo* in an iterative systems biology cycle to better understand the dynamics of infection and the mechanisms of immunoregulation underlying mucosal immune responses to *C*. *difficile*.

The computational model described captures the roles and dynamics of multiple immune and epithelial cell types at the colonic mucosa during the course of CDI (Fig 3). The validated model enables the initial testing of interventions or modulation hypotheses aimed at the improvement of the understanding or treating the CDI. This model could also be used as a tool in determining crucial time points for data collection to maximize the utility of specific pre-clinical and clinical studies. The combined effect of initial testing and targeted time points may greatly improve the cost-efficiency of wetlab experimentation. The model replicates other previously reported experimental results. For instance, treatment with anti-Gr-1 reduces both neutrophil and monocyte levels in the context of a mouse model of *C*. *difficile* infection [31]. However, the reduction does not result in a large change in either the *C*. *difficile* population or disease severity, which also can be shown through varying parameter values and the resulting neutrophil and monocyte levels *in silico*. The association between antibiotic use and recurrence of CDI has been described clinically [28]. Following a simulated reduction of all bacterial species after the initiation of the time course study, the clearance of *C*. *difficile* is incomplete and results in the occurrence of a second peak at a time beyond when complete resolution would occur without intervention. Together with our own generated data, these published experiments serve to verify and validate the ability of the computational model to replicate an *in vivo* infection and produce reliable predictions.

The presence of a robust pro-inflammatory mucosal immune response following CDI at the colonic mucosa is a crucial determinant of the resulting severity of infection. Sensitivity analyses demonstrate that the induction of a regulatory response decreases damage to the epithelium following CDI (Fig 4). Specifically, parameters controlling the production of tolerogenic DC and the plasticity of Th17 cells to become Treg cells are highly associated with decreased epithelial damage. However, the induction of regulatory responses can also slow *C*. *difficile* clearance and increase the overall length of infection, as the removal of pro-inflammatory cell types is largely associated with a larger *C*. *difficile* population. In sensitivity analyses, parameters relating to the production of activated dendritic cells show a large effect in comparison to other parameters. While this may be partially due to the sequential nature of the dendritic cell section of the model network, further investigation of this behavior could be very insightful and immunologically relevant. In many cases, *C*. *difficile* acts as a non-invasive pathogen and the resultant damage is toxin-mediated [32]. However, the epithelium, after damage by the toxins, is still breached, often by bacterial species present in the healthy microbiome that do not normally exhibit pathogenic behavior [33, 34]. Toxin-mediated activation of immune cells occurs, but dendritic cells could potentially exert control over the reactivity to non-*C*. *difficile* infiltrating bacteria. From the high sensitivities in model analysis, the relative ability or inability of dendritic cells or mononuclear phagocytes to maintain tolerogenic responses to commensal bacteria may contribute to the variation in disease severity between patients. Specifically, dendritic cells appear to act as the main drivers of establishing the Th17 response during the peak of infection that controls the pathogenic bacterial population, while also acting as a crucial element in the switch to an iTreg-dominated state during resolution. Similar impact of dendritic cells could be seen in other infections that cause broad damage to the epithelium or are a result of a non-invasive pathogen. Indeed, CX_3_CR1+ mononuclear phagocytes, a population with similar functions to macrophages and conventional dendritic cells, has been shown to prevent reactions to commensal bacteria via driving the differentiation of innate lymphoid cells into subclasses that promote maintenance of barrier integrity and intestinal homeostasis. Recently, these populations have been implicated in the prevention of disease in the context of inflammatory bowel disease and *H*. *pylori* [35]. The use of computational modeling in precision medicine may allow for the determination of a proper balance between pro-inflammatory and regulatory arms of the mucosal immune response in the context of new therapeutic interventions.

CDI is associated with the use of broad-spectrum antibiotics, and microbiome reconstitution through fecal transplantation or probiotic treatment has gained some traction with varying degrees of success [36, 37]. The unspecific nature of these treatments results in the proliferation of a spectrum of *Bifidobacterium* that promotes tolerance to commensal species. However, the *C*. *difficile* population dynamics may be unchanged or poorly altered as a result. Among other methods of inhibition, the growth of *C*. *difficile* has been shown to be slowed by the presence of secondary bile acids [38, 39]. Recently, specific commensal species, that convert primary bile acid to secondary bile acids, have displayed a protective ability against *C*. *difficile* infection [9, 40]. However, the presence of *C*. *difficile* indirectly inhibits the regrowth of these commensal species. Anti-microbial peptides DefB1 and S100A8 are upregulated in colons of *C*. *difficile*-infected mice at distinct time points during infection (Fig 5). DefB1, the gene responsible for beta-defensin-1 produced largely by epithelial cells, is upregulated early and for an extended period in the time course between days 2 and 4 post-infection [41]. While beta-defensin-1 has broad membrane lysis ability, it has its largest effect on gram-negative bacteria [42]. Notably, *C*. *scindens*, a highly prevalent member of bile acid inducible operon containing microbiome, is gram-negative. The combination of elevated beta defensin-1 and the susceptibility of a major constituent of the beneficial microbiome to this anti-microbial peptide suggest a strong probability that the host response to *C*. *difficile* is also inhibitory to the regrowth of beneficial commensal strains. In contrast, expression of S100A8, a component of calprotectin sourced mainly from neutrophils and monocytes, is elevated with a distinct peak on day 4 corresponding with the peak of neutrophil activation [43]. The combined anti-microbial activity may contribute to decreased efficiency in the regrowth of the native microbiome during CDI suggested by computational experimentation (Fig 6). The simulated removal of epithelial cell-related inhibition of commensal bacterial regrowth in the model allows for an increased amount of beneficial commensal species early in the infection, resulting in slightly decreased *C*. *difficile* levels through direct competitive effects on the population itself and indirectly through down-regulation of neutrophil and Th17 cell-mediated effector responses. The alterations within the *C*. *difficile* population during this simulation would be unlikely to significantly affect the initial establishment of a clinical cure through clearance of *C*. *difficile* [44]. However, the lack of effect is still an important result as it suggests that the simulated changes do not result in an inhibition of *C*. *difficile* clearance or a worsening of the hypothetical prognosis. Additionally, the increased and quicker re-growth of commensal bacteria would likely exhibit greater direct effects on the *C*. *difficile* population through the prevention of relapse. The lack of microbial diversity is a major predictor of relapse susceptibility [45]. The return of this diversity would greatly reduce the capacity for the persisting *C*. *difficile* population to expand back to pathogenic levels. During the course of the initial infection, the effect of continued commensal re-growth is largely immunomodulatory rather than displaying a large effect on the *C*. *difficile* population. Continued commensal re-growth throughout the time course displays an ability to shorten the length of CDI and decrease the overall magnitude of the inflammatory host response at the colonic mucosa and resultant collateral damage. This suggests a need for a microbiome reconstitution intervention to restore beneficial effects in terms of the *C*. *difficile* growth inhibition and regulation of a pro-inflammatory environment. The ability to further increase the specificity and success of microbiome reconstitution therapies can be aided by computational modeling.

Neutrophilic influx is a major cause of symptom severity as indicated by sensitivity analysis of our model (Fig 4) and previously reported connections [46, 47]. Subsequently, the chemokine, IL-8, has been studied as a potential marker for severity and prognosis in the diagnosis of *C*. *difficile* infection [48]. In addition to IL-8, other promising biomarkers of *C*. *difficile* associated disease severity include the antimicrobial peptide calprotectin, hepatocyte growth factor, and procalcitonin [49–51]. A future direction in the analysis and application of multiscale models of CDI could be the determination of a non-intuitive vital node in the network; the latter may allow for the extrapolation of easily measurable biomarkers of disease in the context of data-driven models and multiscale models of mucosal immune responses [52]. Consequently, the discovered marker could indicate the stage of infection or the need for or extension of treatment. Methods have been described based on the correlational evaluation of model quantities in virtual populations and global sensitivities in the analysis and discovery of potential biomarkers in the context of lipoprotein metabolism and acute inflammation, respectively [53, 54]. The sensitivity analysis allows for the determination model parameters that are most impactful on immune response and infection outcome. Both methods illustrate the difficulty in the determination of biomarkers without the aid of computational modeling due to the inherent variation of expression levels between individuals. As we have demonstrated the potential changes in mucosal immune response following modifications to the microbiome composition, measurement of beneficial commensal bacterial metabolites, such as deoxycholate and lithocholate, in combination with the computational modeling simulations could help predict the effectiveness of fecal microbiota transplantation and other therapeutic or prophylactic interventions on a patient-to-patient basis or prove to be an effective predictor of untreated outcome. The generation of a synthetic population through a random sampling of parameter values within a normal distribution of the calibrated values would allow for these predictions to be extended into *in silico* clinical trials of CDI.

The model developed and analyzed in this article was specific for the host response to *C*. *difficile*; however, many of the strategies used for this purpose can be extended to better the understanding the responses to other enteric pathogens and inflammatory conditions. Specifically, while fecal transplantation and microbiome reconstitution may be more greatly established in the context of CDI, the concept of promoting the growth of beneficial bacteria could have wide reaching implications in the understanding of mucosal immunity. Currently, there is an increasing prevalence of auto-immune and auto-inflammatory diseases, especially within developed countries [55]. One hypothesis is that improved hygiene and the resultant reduction in microbial exposure have led to decreased regulation of the immune system and an over-exuberant response when an exposure does occur [56]. Additionally, the ability of the host to generate an accommodating environment for commensal species, such as through the secretion of polysaccharides, has been shown to affect the susceptibility to disease [57, 58]. Through the creation of a computational model describing the interactions of bacterial families with the epithelium and elements of the mucosal immune system, an enhanced understanding of which species contribute to the susceptibility and severity of the response could be generated. Furthermore, this increase in knowledge could contribute to the development of targeted pro-biotic and reconstitution therapies to combat the rising rates of auto-immune and auto-inflammatory disease and altered microbiomes resulting from genetic and environmental differences.

In conclusion, we have examined the time course of the immune response to *C*. *difficile* infection through a combination of experimental and computational approaches using an iterative systems biology cycle. We described the importance of maintaining a balance between effector and regulatory arms of the mucosal immune response in the clearance of pathogen and severity of infection. Specifically, the production of anti-microbial peptides may exacerbate disease and pathology, and prolong the CDI due to non-specific inhibition of commensal bacterial regrowth. Computational simulations supported the role of this inhibition on the disease severity with a virtual removal of neutrophil and epithelial cell derived anti-microbial inhibitions, separately and together, on commensal bacterial regrowth. The simulated shifts in host response behavior provide novel insights underlying the mechanisms of interaction between the mucosal immune system and the gut microbiota during CDI.

## Methods

### Ethics Statement

All experimental procedures were approved by the Institutional Animal Care and Use Committee (IACUC) of Virginia Tech and met or exceeded requirements of the Public Health Service/National Institutes of Health and the Animal Welfare Act. The IACUC approval ID for the study was 12-173-VBI. C57BL/6J wild type mice were bred and maintained in experimental facilities at Virginia Polytechnic Institute and State University. Mice were housed two to five per cage on a ventilated rack in a room with a standard 12 hours on, 12 hours off light cycle. The animals were given ad libitum access to standard rodent chow and water. After infection, mice were monitored daily for signs of disease severity and weighed. Four hour checks were triggered when an animal reached a score of three. Mice were euthanized prior to scheduled end point if severe signs of illness, such as a large weight loss, piloerection or a loss of mobility, were present. All mice were euthanized with carbon dioxide narcosis and a secondary cervical dislocation.

### *C*. *difficile* Animal Model

This study followed a previously reported model of *Clostridium difficile* infection [59]. Prior to bacterial challenge, mice were treated with a mixture of antibiotics in drinking water. The mixture consisted of colistin 850 U/mL (4.2 mg/kg), gentamycin 0.035 mg/mL (3.5 mg/kg), metronidazole 0.215 mg/mL (21.5 mg/kg), and vancomycin 0.045 mg/mL (4.5 mg/kg). Mice were kept on the antibiotic water for a three day period corresponding to days 5 to 3 prior to challenge. The mice were returned to standard autoclaved water two days before challenge. The mice were given an intraperitoneal injection of clindamycin, 32 mg/kg, one day prior to infection. The control group received the same antibiotic pretreatment. The infected group was challenged through intragastric gavage with *Clostridium difficile* strain VPI 10463 (ATCC 43255) 10^7^ cfu in 200 uL/mouse of Brucella broth. Mice were weighed and scored daily to assess the presence of disease symptoms (diarrhea, piloerection, hunchback position, etc.). Mice were sacrificed through CO_2_ narcosis and secondary cervical dislocation at different time points (days 1, 3, 4, 5, 7, 8, 10) post infection.

### Sample Processing

Mesenteric lymph nodes and colons were collected. Mesenteric lymph nodes (MLN) were crushed using the frosted ends of microscope slides. Samples were centrifuged and washed with phosphate buffered saline (PBS) containing 5% fetal bovine serum (FBS) and Golgi stop. Cells were centrifuged and re-suspended in FACS buffer and counted using BD Coulter cell counter. Colon samples were washed in BD Cell Recovery Media to remove epithelial cells. Remaining tissue was degraded in RPMI containing collagenase and DNase at 37°C while stirring. Samples were filtered using and centrifuged. Remaining cells were re-suspended and purified in a Percoll gradient. Cells at the Percoll interface were collected and counted.

### Flow cytometry

MLN and colonic lamina propria lymphocytes were plated in 96 well plates (6x10^5^ cells/well). Cells were incubated with fluorochrome conjugated antibodies to extracellular markers, anti-CD45 APC-Cy7, anti-CD3 PE-Cy5, anti-CD4 PE-Cy7, anti-CD25 biotin, anti-CD64 PE, anti-CD11b AlexaFluor700, anti-F4/80 PE-Cy5, anti-CD11c FITC, anti-Gr1 PE-Cy7, anti-Ly6c PerCP-Cy5.5, and anti-MHC-II biotin. Samples needing a secondary staining were incubated with Streptavidin-Texas Red. The samples were then fixed and permeabilized. Cells were incubated with antibodies to intracellular markers, anti-FoxP3 FITC, anti-IL-10 APC, anti-RORγT PE, and anti-IL-17 APC. Data was acquired with a BD LSRII flow cytometer and analyzed using FACS Diva software (BD Pharmingen).

### Bacterial re-isolation

Colonic contents were collected from excised colons. Samples were homogenized in Brucella broth and incubated at 68°C for one hour. Samples were centrifuged at 10,000 rpm for 30 seconds and the supernatant was collected. The supernatant was serially diluted (1:10, 1:100, 1:1000) and plated on Oxoid *Clostridium difficile* agar plates containing *Clostridium difficile* selective supplement. Plates were incubated in anaerobic conditions using a BD EZ anaerobic container system kit for 2 days at 37°C. Colonies were counted and compared to sample weight for normalization.

### Gene expression

Total RNA was isolated from mouse colonic contents using a Qiagen RNA isolation mini kit. Complementary DNA (cDNA) was generated from each sample using the iScript cDNA synthesis kit. Standards were produced through a polymerase chain reaction on the cDNA with Taq DNA polymerase from Invitrogen. The amplicon was purified using the Mini-Elute PCR purification kit from Qiagen. Expression levels were obtained through quantitative real-time PCR on a Bio-Rad CFX 96 Thermal Cycler using the Bio-Rad SYBR Green Supermix. For analysis, the starting amount of anti-microbial peptide cDNA was compared to that of beta-actin, as a control. Primer sequences are provided in supplemental information (S3 Table).

### Computational modeling

The generation of a computational model was used in combination with experimental methods to improve our understanding of gathered data, to create a more systematic experimental process and to generate new knowledge. The model generation was a multi-step process, including the creation of a model network, calibration and validation of the model equations, analysis of the model, and execution of *in silico* simulations. The structure of the computational model, which includes the species and their interactions, was constructed in CellDesigner, a Systems Biology Markup Language (SBML) compliant software. The network was generated based on a combination of generated time course data and a thorough literature review and depicts the cellular host involving interactions between dendritic cells, T helper cells, macrophages, neutrophils, epithelial cells and commensal bacteria. The model was imported into Complex Pathway Simulator (COPASI) software[27]. In COPASI, the interactions and transitions were assigned ordinary differential equations representing multiple kinetics including mass action, simple activation and Hill-type activation and inhibition, available in supplemental information (S1 File). The resulting parameters were estimated using Particle Swarm and Genetic algorithms with time course data generated through the mouse model of infection on days 1, 4, 7 and 10 in addition to extra days post-infection included in sourced data. The parameter search algorithms seek to minimize the sum of squares for the calibration dataset. To further train the model, a separate dataset set containing data from days 3, 5, and 8 post-infection was used as a validation dataset. In the parameter estimation process, the sum of squares for the validation dataset is monitored but not minimized. Rather an increase in the sum of squares for the validation dataset is used as a stop criterion for the search algorithm which serves as a preventative measure against over-fitting. Parameters values and further information on the parameter fitting process are available in supplemental information (S1 Table and S2 File). Time course simulations were conducted using an LSODA deterministic method. The model displays the ability to represent activation, differentiation and death of the cell types involved and allows for distinctions to be made in patterns and sequences of events. Local sensitivities were calculated through numerical differentiation using a finite difference method with delta factor 0.001 and delta minimum 1x10^-12^. The sensitivity analysis was used to elucidate the combination of direct and indirect effects. Furthermore, all model quantities had on specific outcome events which allows for the identification of potential target nodes with which a desired change can be induced. *In silico* simulations on the effects of anti-microbial inhibition of commensal regrowth were conducted through modification of the CommB to CommD transition. The resulting changes were observed in a time course simulation. The full model is deposited in the Biomodels Database (https://www.ebi.ac.uk/biomodels-main/) with identifier MODEL1507200000.

### Model assumptions

Initial particle numbers are assumed to be representative of a post-antibiotic ablation state in which commensal species are greatly reduced. The model requires an initial amount of the *Cdiff* species to be present for the response to be initiated. Reactions may represent direct cell-to-cell contact (sensing of *C*. *difficile* by dendritic cells), cytokine- or toxin-mediated effects (the differentiation and activation of CD4+ T cell populations), or cellular movement (migration of T cells from the mesenteric lymph nodes to the lamina propria). Cross-compartmental reactions are possible through the environmental changes induced by the effector cell in the reaction. The model assumes that protective commensal species follow a regrowth pattern similar to a summation of *Clostridiaceae*, *Ruminococceae*, *Verrucomicrobiaceae*, *Porphyromonadaceae*, *Turicibacteraceae*, and *Eubacteriaceae* bacterial families, while the infection-exacerbating population is assumed to follow that of *Enterobacteriaceae*, *Streptococcaceae*, and *Enterococcaceae* families. Reactions in the model may be simplifications of multi-step processes. Non-informative parameters were eliminated from the model through simple deletion of the reaction or fusion with a related neighboring reaction. Parameters were deemed non-informative through local sensitivity analysis within COPASI using numerical differentiation through finite differences. For instance, the initial model contained separate steps for the activation and migration of neutrophils. Because the sensitivity to the migration parameter was five orders of magnitude less than the sensitivity to the activation parameter, the reaction corresponding to the migration was combined into the activation reaction.

### Statistical analysis

A one way analysis of variance (ANOVA) was performed to determine significance in the data using a SAS (SAS Institute) general linear model procedure. Differences of p≤0.05 were considered significant. Data was comprised of multiple experiments. The number of samples per for each group at each time point varied between five and eight. Data is displayed as mean values with error bars representing standard error of the mean and asterisks to mark significance.

## Supporting Information

S1 File Ordinary differential equations of model. Equations control the dynamics of the computational model. Mass action, simple activation/inhibition and Hill-type activation/inhibition were used in the generation of the equations.
(PDF)

S2 File Description of parameter fitting process. The file provides more in-depth discussion of parameter fitting to complement the summary provided in Methods.
(PDF)

S1 Table Parameters values. Table contains the calibrated parameter values of the model which were generated through Particle Swarm and Genetic algorithms parameter estimation methods in combination with experimentally obtained data.
(XLSX)

S2 Table Calibration and validation databases for estimation of model parameters. Database includes newly generated and previously reported time course data for the response to *C*. *difficile* infection.
(XLSX)

S3 Table Primers used to characterize commensal species and anti-microbial peptide expression. Primers cover the *baiCD* operon in bacterial strains and the anti-microbial peptide associated genes DefB1 and S100A8.
(XLSX)
