# Supplementary material for: Systems Modeling of Interactions between Mucosal Immunity and the Gut Microbiome during Clostridium difficile Infection
Source: PLoS One. 2015 Jul 31;10(7):e0134849. doi: 10.1371/journal.pone.0134849 (PMC4521955; doi:10.1371/journal.pone.0134849)
Supplement: S1 File — Equations control the dynamics of the computational model. Mass action, simple activation/inhibition and Hill-type activation/inhibition were used in the generation of the equations. (PDF) [file pone.0134849.s001.pdf]

1. $\frac{d[Cdiff]}{dt} = \frac{K_{CG}[Cdiff][CH]}{[CB]} - K_{tDC}[Cdiff]\left(\frac{\gamma_{tDC1}[CB]}{[CoD]} + \frac{\gamma_{tDC1}[E]}{[Ei]+\beta}\right) - K_{eDC}[Cdiff] - d_{Cd}[Cdiff]([M_{LP}] + \gamma_{Cd}[N_{lum}] - \alpha_{Cd}[CH])$

2. $\frac{d[N_{lum}]}{dt} = K_{NAM}[N_{LP}][Cdiff](\gamma_{NAM1}[Ed] + \gamma_{NAM2}[Th17_{LP}] - \alpha_{NAM}[Treg_{LP}]) - d_N[N_{lum}][CB]$

3. $\frac{d[CB]}{dt} = -K_{CR1}[CB][N_{lum}][Ei] + K_{CR2}[CoD]$

4. $\frac{d[M_{LP}]}{dt} = K_{MA}[M0]([Cdiff] + \gamma_{MA}[Th17_{LP}] - \alpha_{MA}[Treg_{LP}]) - d_M[M_{LP}]$

5. $\frac{d[CD]}{dt} = K_{CR1}[CB][N_{lum}][Ei] - K_{CR2}[CoD] - K_{CoD}[CoD]$

6. $\frac{d[tDC_{LP}]}{dt} = K_{tDC}[Cdiff]\left(\frac{\gamma_{tDC1}[CB]}{[CoD]} + \frac{\gamma_{tDC2}[E]}{[Ei]+\beta}\right) - \sigma_{tDC}[tDC_{LP}]$

7. $\frac{d[tDC_{MLN}]}{dt} = \sigma_{tDCM}[tDC_{LP}] - d_{tDC}[tDC_{MLN}] - K_{Tr}[tDC_{MLN}]$

8. $\frac{d[CH]}{dt} = -d_{CH}[CH]$

9. $\frac{d[E]}{dt} = K_{EH}[Ed] - K_{EI}[E][Cdiff] - d_E[E](\gamma_{ED1}[N_{lum}] + \gamma_{ED2}[Th17_{LP}] + \gamma_{ED3}[M_{LP}])$

10. $\frac{d[Ed]}{dt} = d_E[E](\gamma_{ED1}[N_{lum}] + \gamma_{ED2}[Th17_{LP}] + \gamma_{ED3}[M_{LP}]) - K_{EH}[Ed] + d_{EN}[Ei] + d_{Ei}[Ei](\gamma_{EiD1}[N_{lum}] + \gamma_{EiD2}[Th17_{LP}] + \gamma_{EiD3}[M_{LP}])$

11. $\frac{d[Ei]}{dt} = K_{EI}[E][Cdiff] - d_{Ei}[Ei](\gamma_{EiD1}[N_{lum}] + \gamma_{EiD2}[Th17_{LP}] + \gamma_{EiD3}[M_{LP}]) - d_{EN}[Ei]$

12. $\frac{d[eDC_{LP}]}{dt} = K_{eDC}[Cdiff] - \sigma_{eDC}[eDC_{LP}]$

13. $\frac{d[Th17_{LP}]}{dt} = -\mu_{T1}[Th17_{LP}] + \mu_{T2}[Treg_{LP}][Cdiff] + \sigma_{T17}[Th17_{MLN}] - d_{T17}[Th17_{LP}]$

14. $\frac{d[Th1_{LP}]}{dt} = \sigma_{T1}[Th1_{MLN}] - d_{T1}[Th1_{LP}]$

15. $\frac{d[Treg_{LP}]}{dt} = \mu_{T1}[Th17_{LP}] - \mu_{T2}[Treg_{LP}][Cdiff] + \sigma_{Tr}[Treg_{MLN}] - d_{Tr}[Treg_{LP}]$

16. $\frac{d[eDC_{MLN}]}{dt} = \sigma_{tDC}[tDC_{LP}] - K_{T1}[eDC_{MLN}]\left(\frac{[CD]}{\alpha_{T1C}[CB]+\alpha_{T1E}[E]}\right) - K_{T17}[eDC_{MLN}] - d_{eDC}[eDC_{MLN}]$

17. $\frac{d[Treg_{MLN}]}{dt} = K_{Tr}[tDC_{MLN}] - \sigma_{Tr}[Treg_{MLN}]$

18. $\frac{d[Th17_{MLN}]}{dt} = K_{T17}[eDC_{MLN}] - \sigma_{T17}[Th17_{MLN}]$

19. $\frac{d[Th1_{MLN}]}{dt} = K_{T1}[eDC_{MLN}]\left(\frac{[CD]}{\alpha_{T1C}[CB]+\alpha_{T1E}[E]}\right) - \sigma_{T1}[Th1_{MLN}]$

The list of nineteen differential equations defines the dynamics of the model. One equation exists for each species (denoted by the bracketed, [ ], terms in the above equations) in the model network shown in Fig. 1 of the main text. More than one reaction may be encompassed in each equation. For example, the differential equation for [Cdiff] includes terms pulled from four reactions (Cdiff Death, Cdiff Growth, tDC production and eDC production). The terms can be mapped to their parent reaction through the subscript and parameter type. A full description of the parameters and their calibrated values is provided in supplemental information (S1 Table). The following table provides a key to parameter notation.

Parameter	Function
K	production, activation and differentiation
d	damage, death and degradation
σ	migration
γ	activation
α	inhibition
β	denominator constant
μ	plasticity
