# Supplementary material for: Systems Modeling of Interactions between Mucosal Immunity and the Gut Microbiome during Clostridium difficile Infection
Source: PLoS One. 2015 Jul 31;10(7):e0134849. doi: 10.1371/journal.pone.0134849 (PMC4521955; doi:10.1371/journal.pone.0134849)
Supplement: S2 File — The file provides more in-depth discussion of parameter fitting to complement the summary provided in Methods. (PDF) [file pone.0134849.s002.pdf]

Description of parameter fitting process

The unknown parameters within the computational model derived from the network topology (Fig.1) were fit using the parameter estimation feature of COPASI [1], which is able to fit the SBML compliant model to multiple experiments simultaneously. We used 2 global optimization algorithms: Particle Swarm[2] and Genetic algorithms [3] provided within COPASI . The search algorithms attempt to minimize the sums of squares of the variation between the provided experimental data, termed the calibration dataset, and the simulated dynamics. The Particle Swarm method functions through creating a number of parameter sets based on the initial guess. Each of these sets is given a position and velocity. During each iteration of the method, the sets generate a new velocity based on the comparative fit of itself to its neighboring sets. The best optimization of the sum of squares is kept in memory. The Genetic algorithm creates a population of parameter sets. These parameter sets reproduce by combining traits with another set to create a hybrid set. During each round of the algorithm, the sets compete and the highest performing sets survive the reduction to the original number of sets. For both algorithms, the process is repeated until the fit to the calibration dataset is optimized or the maximum number of iterations (2000 iterations) is reached. In both cases, a second dataset, termed the validation dataset, is used during the parameter fitting process. The sum of squares for the validation dataset is monitored but not minimized. Rather an increase in the sum of squares for the validation dataset is used as a stop criterion for the search algorithm which serves as a preventative measure against over-fitting. Parameters values are available in supplemental information (S1 Table).

The parameter fitting was carried out with time course data generated through the mouse model of infection on days 1, 4, 7 and 10 in addition to extra days post-infection included in data sourced from other publications. To further train the model, a separate dataset set containing data from days 3, 5, and 8 post-infection was used as a validation dataset. The parameter fitting was conducted by using data in triplicate for each time point. For example, the $Th17_{LP}$ variable had 21 total data points inputted for the parameter fitting process. Twelve of these points (three replicates each on days 1, 4, 7, and 10) were used for the calibration dataset and nine were used in the validation data set. Average values for every data point used within the parameter fitting process are included with supplemental information (S2 Table).

The calibration process was conducted on a global scale. Therefore, all parameters were allowed to vary to fit the experimental data. As the model contains a large number of parameters, calibrating the entire model in one step would not provide an accurate or desirable fit. To accomplish the global calibration, an iterative strategy was used in which the model was divided into five subsections each of which was calibrated individually. These sub-sections were: microbial (Cdiff, CommH, CommB, and CommD species), epithelial (E, Ei, Ed), T cells ($Th17_{LP}$, $Th1_{LP}$, $Treg_{LP,}$ $Th17_{MLN}$, $Th1_{MLN}$, $Treg_{MLN}$), dendritic cells (eDC_{LP}, tDC_{LP}, eDC_{MLN}, tDC_{MLN}), and macrophage/neutrophil (M0, M_{LP}, N_{LP}, N_{Lum}). After each of the subsections was able to capture the dynamics, the model was assembled to its original state and re-calibrated with all interactions in place. The parameter values obtained from the subsection calibrations were used as initial estimates in this stage of the process. Each parameter was allowed to vary within a range 10% above or below the initial value according to upper and lower bounds set during the search process. Each search algorithm was run a minimum of five times. The final parameter set was chosen by comparison of objective values, root mean squares, and standard deviations generated in different runs of the calibration.

References

1. Hoops S, Sahle S, Gauges R, Lee C, Pahle J, et al. (2006) COPASI--a COmplex PAthway SImulator. Bioinformatics 22: 3067-3074.
2. Kennedy J, Eberhart R. Particle swarm optimization; 1995. IEEE. pp. 1942-1948.
3. Michalewicz Z (1995) Genetic algorithms, numerical optimization and constraints. International Conference on Genetic algorithms.
